# Auto-segmentation of neck nodal metastases using self-distilled masked image transformer on longitudinal MR images

**DOI:** 10.1093/bjrai/ubae004

**Published:** 2024-03-04

**Authors:** Ramesh Paudyal, Jue Jiang, James Han, Bill H Diplas, Nadeem Riaz, Vaios Hatzoglou, Nancy Lee, Joseph O Deasy, Harini Veeraraghavan, Amita Shukla-Dave

**Affiliations:** Department of Medical Physics, Memorial Sloan Kettering Cancer Center, New York, NY 10065, United States; Department of Medical Physics, Memorial Sloan Kettering Cancer Center, New York, NY 10065, United States; Department of Radiation Oncology, Memorial Sloan Kettering Cancer Center, New York, NY 10065, United States; Department of Radiation Oncology, Memorial Sloan Kettering Cancer Center, New York, NY 10065, United States; Department of Radiation Oncology, Memorial Sloan Kettering Cancer Center, New York, NY 10065, United States; Department of Radiology, Memorial Sloan Kettering Cancer Center, New York, NY 10065, United States; Department of Radiation Oncology, Memorial Sloan Kettering Cancer Center, New York, NY 10065, United States; Department of Medical Physics, Memorial Sloan Kettering Cancer Center, New York, NY 10065, United States; Department of Medical Physics, Memorial Sloan Kettering Cancer Center, New York, NY 10065, United States; Department of Medical Physics, Memorial Sloan Kettering Cancer Center, New York, NY 10065, United States; Department of Radiology, Memorial Sloan Kettering Cancer Center, New York, NY 10065, United States

**Keywords:** deep learning, convolutional neural network, neck nodal metastases, auto-segmentation, self-supervised learning, vision transformers

## Abstract

**Objectives:**

Auto-segmentation promises greater speed and lower inter-reader variability than manual segmentations in radiation oncology clinical practice. This study aims to implement and evaluate the accuracy of the auto-segmentation algorithm, “Masked Image modeling using the vision Transformers (SMIT),” for neck nodal metastases on longitudinal T_2_-weighted (T_2_w) MR images in oropharyngeal squamous cell carcinoma (OPSCC) patients.

**Methods:**

This prospective clinical trial study included 123 human papillomaviruses (HPV-positive [+]) related OSPCC patients who received concurrent chemoradiotherapy. T_2_w MR images were acquired on 3 T at pre-treatment (Tx), week 0, and intra-Tx weeks (1-3). Manual delineations of metastatic neck nodes from 123 OPSCC patients were used for the SMIT auto-segmentation, and total tumor volumes were calculated. Standard statistical analyses compared contour volumes from SMIT vs manual segmentation (Wilcoxon signed-rank test [WSRT]), and Spearman’s rank correlation coefficients (*ρ*) were computed. Segmentation accuracy was evaluated on the test data set using the dice similarity coefficient (DSC) metric value. *P*-values <0.05 were considered significant.

**Results:**

No significant difference in manual and SMIT delineated tumor volume at pre-Tx (8.68 ± 7.15 vs 8.38 ± 7.01 cm^3^, *P* = 0.26 [WSRT]), and the Bland-Altman method established the limits of agreement as –1.71 to 2.31 cm^3^, with a mean difference of 0.30 cm^3^. SMIT model and manually delineated tumor volume estimates were highly correlated (*ρ* = 0.84-0.96, *P* < 0.001). The mean DSC metric values were 0.86, 0.85, 0.77, and 0.79 at the pre-Tx and intra-Tx weeks (1-3), respectively.

**Conclusions:**

The SMIT algorithm provides sufficient segmentation accuracy for oncological applications in HPV+ OPSCC.

**Advances in knowledge:**

First evaluation of auto-segmentation with SMIT using longitudinal T_2_w MRI in HPV+ OPSCC.

## Introduction

Cross-sectional modalities, such as computed tomography (CT) and magnetic resonance imaging (MRI), are used for tumor detection and treatment planning.^1–3^ In routine clinical practice, identifying and segmenting the organs at risk (OARs) and tumors on CT imaging is critical to delivering precision radiotherapy.^4^ CT imaging provides a solid geometric and electron density map for accurate dose calculation on the tumor, surrounding tissues, and OARs but suffers from limitations, including artifacts from metals and uncertainty in tumor-normal soft tissue boundaries.^5^ MRI is a noninvasive imaging technique and offers superior soft-tissue contrast to CT, thus emerging as a potential imaging method in radiotherapy (RT) planning, delivery, and treatment assessment.^6^^,^^7^ In addition, quantitative imaging biomarkers (QIBs) obtained from segmented regions of interest (ROIs) on MR images of head and neck cancer (HNC) provide unique insight into underlying tumor physiology.^8–11^ 

Traditionally, radiologists manually delineate the tumor boundaries on CT and MR images in HNC; however, this is a time-consuming process and prone to high inter-reader variability due to the morphological complexity of HNC and potential artifacts.^12^^,^^13^ Accurate delineation of OARs and tumor volumes is critical for minimizing radiation toxicities and maximizing tumor control in HNC’s concurrent chemoradiotherapy (CCRT) planning.^14^ Atlas-based segmentation has proven to be a promising time-saving method for contouring HNC patients undergoing RT or CCRT.^15^ However, manual editing is still needed for small target tumor volumes in the head and neck regions.^16^

Recently, machine learning approaches promise automated segmentation that can address the above challenges,^17^ offering better efficiency and reproducibility than manual and atlas-based segmentation.^18^ Deep learning (DL) tools using convolutional neural networks (CNNs) have also shown potential for contouring OARs^19^^,^^20^ and tumors in the HN region.^21–24^ Representation or self-supervised learning (SSL) is a way to learn the features from the data unsupervised. SSL method includes image inpainting and outpainting,^25^ MedicalNet,^26^ and constractive learning.^27^ Most DL tools are based on supervised learning, where imaging data with manual segmentation is used to train a CNN. The interactive deep-learning method is a different approach that combines the power of CNNs with physicians’ knowledge, reducing the need to train models using carefully curated and labeled datasets.^28^ The image foundation model called Segment Anything, which was developed for 2D natural image analysis, exemplifies this approach applied to medical images.^29^ This prompt tuning method provides inputs like bounding boxes and point clicks within ROI and can produce reasonable segmentations for some organs.^30^

In HNC, Korte et al^20^ demonstrated that cascaded CNNs on T_2_-weighted (T_2_w) MRI images of 31 patients could generate automated, high-resolution parotid and submandibular glands segmentation with improved geometric accuracy. Kawahara et al^19^ investigated the auto-segmentation of the OAR for HNC patients using U-net and Generative Adversarial Networks (GAN) models on 55 sets of HN T_2_w MR images. They found that the 3D-Unet model can improve the efficiency of HN RT treatment planning. Schouten et al^23^ performed auto-segmentation for the primary tumor volume in 220 HNC patients using a Multiview (MV) CNN method on multi-contrast MRI (ie, T_1_w, STIR, and contrast-enhanced T_1_w), yielding a dice similarity coefficient (DSC) of 0.49. Rodríguez Outeiral et al^31^ performed the CNNs segmentation on multi-contrast MRI sequences (ie, T_1_w, T_2_w, and 3D post-contrast T_1_w) on the primary tumor of 171 HNC and showed that 3D Unet combining all multi-contrast MRI sequences performed better than a single contrast sequence (median DSC = 0.55). Wahid et al^24^ used multiparametric (mp) MRI input channels T_2_w, T_1_w, apparent diffusion coefficient, volume transfer constant (K^trans^), and extravascular extracellular volume fraction (v_e_) for 3D Residual U-net modeling in 30 HNC primary gross tumor volume segmentation and found that the combination model with T_2_w and T_1_w MR images achieved a slightly higher DSC = 0.73 than all inputs together (DSC = 0.71).

Emerging literature supports the effectiveness of SSL with vision transformers, which offer the ability to learn from unlabeled data without supervision, providing a novel alternative to CNN for medical image segmentation.^32^^,^^33^ Vision transformers use self-attention mechanisms to capture global dependencies and learn representations directly from raw pixel image data without relying on hand-crafted features.^34^^,^^35^ SSL models can be trained to generate useful representations of images, which can be fine-tuned on specific downstream tasks. Jiang et al^36^ successfully developed self-distillation learning with masked images using a vision transformers (SMIT) foundation model for 3D multi-organ segmentation and validated it on 3,643 clinical imaging examinations. SMIT learns 3 pretext tasks, including global and patch self-distillation learning and pixel-wise image prediction, and performs self-distillation.^36^^,^^37^ Employing a dense pixel-wise regression within masked patches called masked image prediction, combined with masked patch token distillation as a pretext task, the SMIT model exhibited more accuracy and required fewer fine-tuning datasets than other pretext tasks such as CNN Random DSC = 0.798 vs SMIT DSC = 0.875.

The study aims to implement and evaluate the accuracy of SMIT model auto-segmentation for neck nodal metastases on longitudinal T_2_w MR images in human papillomavirus (HPV-positive [+]) related oropharyngeal squamous cell carcinoma (OPSCC) patients.

## Methods

### Patients

Our institutional review board approved a retrospective analysis of the data collected in a prospective dose de-escalation clinical trial. Written informed consent was obtained from all eligible HPV+ OPSCC patients with neck nodal metastases before enrollment in the original de-escalation trial, which studied a 30 Gy dose for radiotherapy and chemotherapies including cisplatin or carboplatin/5-Fluorouracil.^10^ Between February 2018 and December 2020, 123 HPV+ OPSCC patients were enrolled. Demographic and clinical characteristics are given in Table 1.

**Table 1.** Patient characteristics.

Characteristics	*n* (%)
*Age (years)*	
Median	58 (36-80)
*Sex*	
Male	109 (89)
Female	14 (11)
*Clinical stage*	
III	15 (12)
IVa	108 (88)
*Primary tumor location*	
Tonsil	63 (51.2)
BOT	40 (32.5)
Unknown primary	20 (16.3)

**Figure 1.**
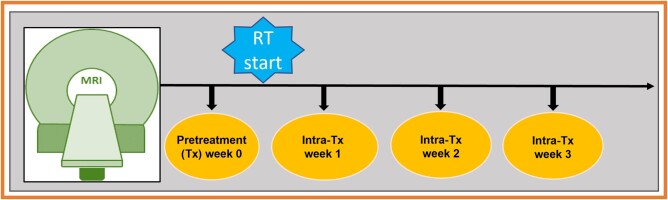
Schema illustrating the timeline for longitudinal MRI examinations.

Longitudinal MRIs were performed at pre-treatment (Tx) (week 0) and intra-Tx weeks (1-3 weeks) during CCRT for the HPV+ OPSCC patients (Figure 1). For our retrospective analysis, 123 HPV+ OPSCC patients’ longitudinal T_2_w MRI data (pre-Tx and intra-Tx weeks (1-3)) were used for manual segmentation.

### MRI data acquisition

Images were acquired on a Philips 3 T scanner (Ingenia; Philips Healthcare, Netherlands) using a neurovascular phased-array coil. The standard MR acquisition comprised multi-planar T_2_w (echo time [TE] = 80 ms, repetition time [TR]/TE = 4099-5939 ms, number of averages [NA] = 2, number of slices [NS] = 50, matrix = 256 × 256, slice thickness = 4 mm, field of view [FOV] = 20-24 cm) and pre-contrast and post-contrast T_1_w images (TR = 681 ms, TE = 8 ms, NA = 2, NS = 40, slice thickness = 4.0 mm; matrix = 256 × 256, FOV = 20-24 cm). The total MRI acquisition time for the standard imaging was ∼30 min.

### Regions of interest contouring

T_2_w images were chosen for contouring based on standard practice in radiation oncology clinics. A team of 2 radiation oncologists with more than 5 years of experience manually contoured the neck nodal metastases on longitudinal T_2_w images using ITK-SNAP.^38^ These manually delineated contours were our estimate of human performance for comparison with the delineation generated by auto-segmentation. Final contoured ROIs were determined in consensus with one neuroradiologist with more than 10 years of experience, using the postcontrast T_1_w images as a reference. These final consensus-contoured ROIs were used as ground truths for the SMIT model’s auto-segmentation. The tumor volumes were calculated from the manually delineated and auto-segmentation ROIs using ITK-SNAP.

### Deep learning-based auto-segmentation method

**Figure 2.**
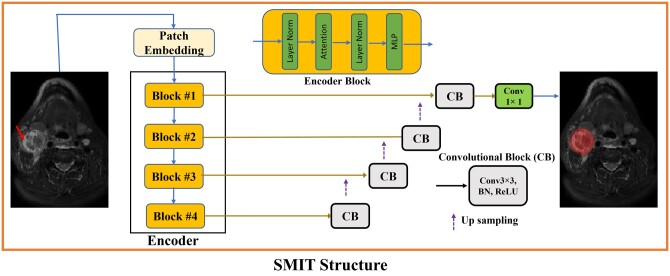
The workflow for the self-distilled masked image transformer (SMIT) model.

Our study employed the SMIT method, which uses the pretrained 3D Swin model in neck nodal metastases on longitudinal T_2_w MRI images.^36^ Herein, the T_2_w MR images of a patient from different treatment time points were grouped together and not treated individually. The pretrained encoder was combined with an Unet decoder and then fine-tuned for this study using the longitudinal T_2_w MRI (Figure 2). The decoder consisted of 4 convolutional layers followed by a SoftMax activation function previously used for generating segmentation of multiple organs from CT and MRI. Out of 123 patients, 95 were employed for training, 10 for validation, and 18 for testing in this study. Training used a patch size of 128 × 128 × 128. During testing, the same patch size, 128 × 128 × 128 pixels, with a sliding window of 0.5, was implemented to segment the image volume.^36^ The segmented results were then resampled to the original voxel size to provide the auto-segmentation for the neck nodal metastases. Five hundred epochs were used for model training in 20 hours, and fine-tuning was performed using 5-fold cross-validation. The average SMIT model inference time is 2 s, including data loading and segmentation.

### Statistical analysis

The tumor volumes obtained with the SMIT model auto-segmentation and manual contouring were reported (mean ± SD). The segmentation accuracy of the SMIT algorithm was assessed using DSC. The Wilcoxon signed-rank test (WSRT) was utilized to compare tumor volumes delineated with the SMIT model and manual and DSC metric values between the treatment time points. Spearman’s rank correlation coefficients (*ρ*) were computed for tumor volume estimates with the SMIT model and manual delineation and DSC. The final consensus-contoured ROIs served as the ground truth. Bland-Altman analysis assessed the agreement between the 2 methods of tumor volume measurements. A *P*-value <0.05 was considered statistically significant.

## Results

The longitudinal T_2_w MRI datasets (neck nodal metastases) from 123 patients at pre-Tx and intra-Tx weeks 1, 2, and 3 were used in this retrospective autosegmentation. For the SMIT algorithm, 95, 10, and 18 patient datasets were utilized to train, validate, and test among these patients.

**Figure 3.**
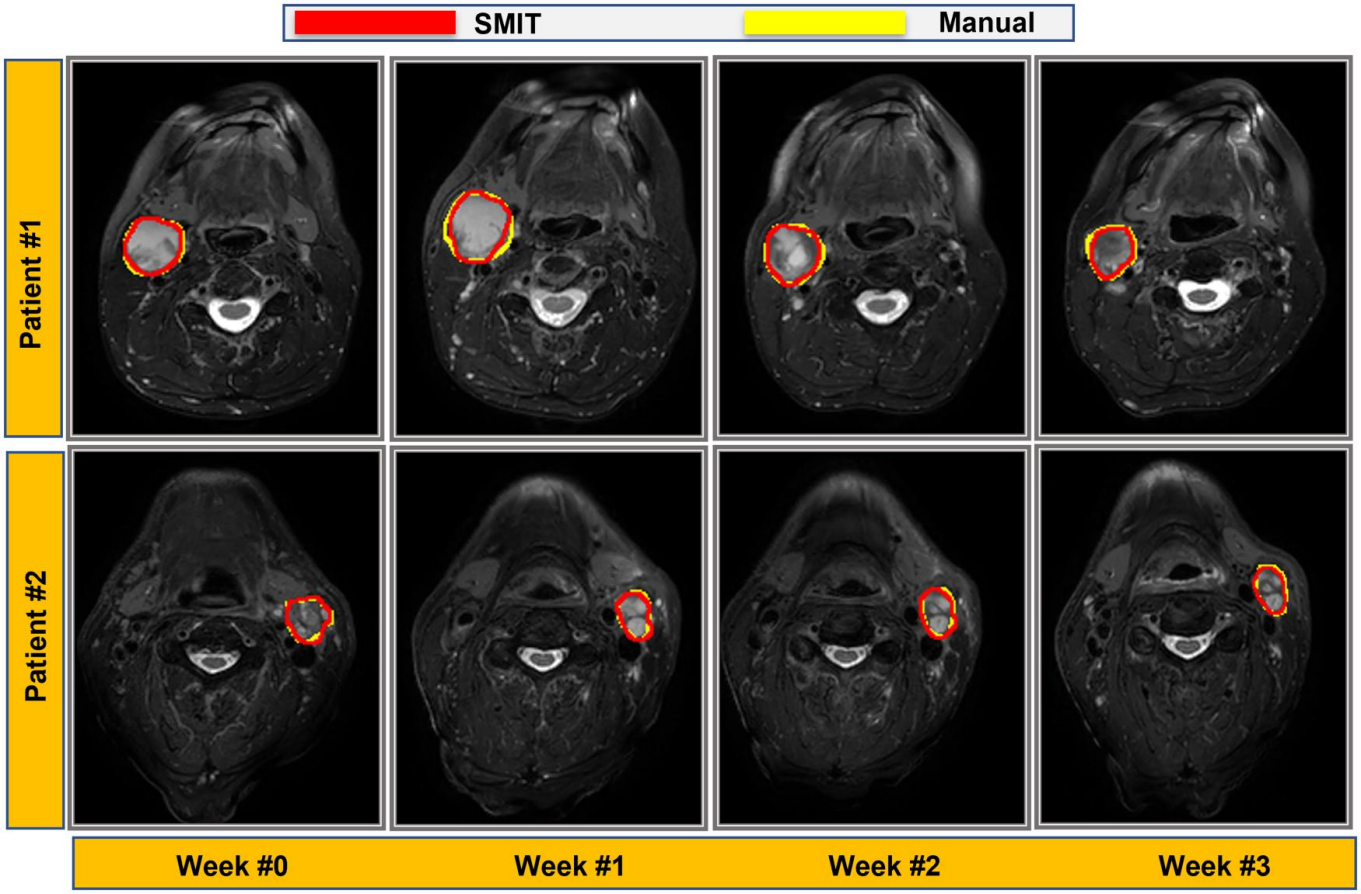
Representative auto-segmentation with self-distilled masked image transformer (SMIT) model auto-segmentation and manually contoured neck nodal metastases on longitudinal T2w MR images from 2 representative oropharyngeal squamous cell carcinoma patients (Patient #1, 41-year-old male and Patient #2, 57-year-old male). The SMIT model generated contours that agreed with ground truth contours across time.

Two representative longitudinal metastatic neck nodal tumor volumes segmented manually (outlined in yellow) and with SMIT (outlined in red) at pre-Tx and intra-Tx weeks 1-3 from HPV+ OPSCC patients are illustrated in Figure 3. For patient #1, the tumor volumes from manual delineation and auto-segmentation were 5.46 vs 5.06 cm^3^ at pre-Tx (week 0), 8.54 vs 6.60 cm^3^ at week 1, 5.36 vs 4.88 cm^3^ at week 2, and 5.06 vs 4.35 cm^3^ at week 3, respectively. For patient #2, the manual delineation vs auto-segmented tumor volumes were 8.51 vs 8.14 cm^3^ at week 0, 14.94 vs 14.60 cm^3^ at week 1, 8.99 vs 8.31 cm^3^ at week 2, and 17.70 vs 18.71 cm^3^ at week 3. The SMIT method exhibited concordance with ground truth for the neck nodal metastases of the 2 representative HPV+ OPSCC patients.

**Figure 4.**
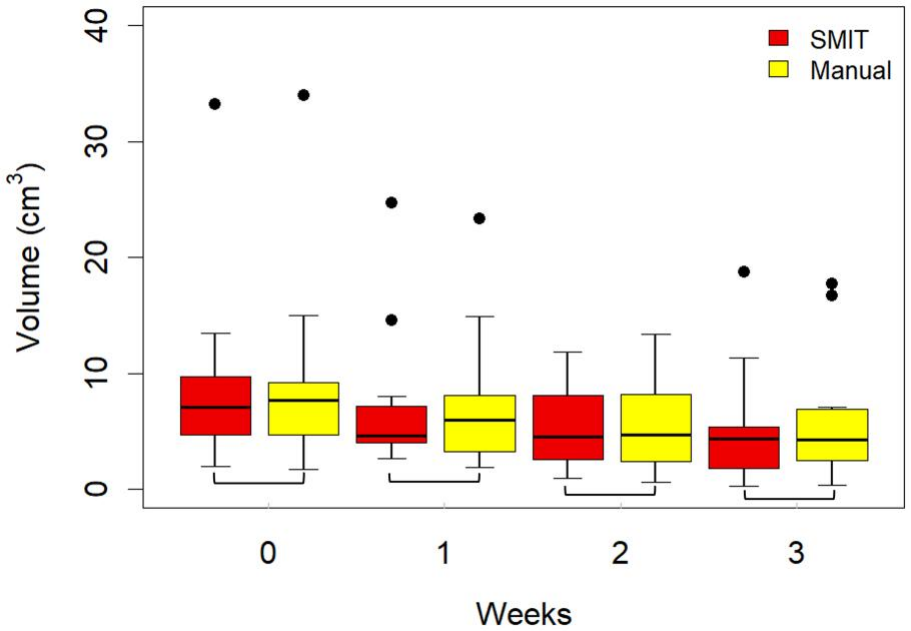
Box and whisker plot shows the mean neck nodal total metastases tumor volumes from self-distilled masked image transformer (SMIT) model auto-segmentation and manual contouring at pre-Tx and intra-Tx weeks (1-3) in oropharyngeal squamous cell carcinoma patients. SMIT and manual contouring total tumor volumes were not significantly different (*P* > 0.05). The horizontal line within each box represents the median value (black line). The black close circle represents the outliers.

Longitudinal metastatic neck nodal tumor volumes obtained by the SMIT model were not significantly different from manual contouring (WSRT, *P* > 0.05) (Table 2, Figure 4), exhibiting a strong agreement between the 2 approaches. The pre-Tx volumes with SMIT and ground truth (M) were 8.38 ± 7.01 vs 8.68 ± 7.15 cm^3^, the change in volume (ΔV_M-SMIT_) = 0.30 cm^3^ compared to intra-Tx week 3, 5.24 ± 5.39 vs 6.14 ± 5.82 cm^3^, Δ_VM-SMIT_ = 0.90 cm^3^. The total tumor volume from manual delineation was significantly correlated with the SMIT model (*ρ* = 0.84 to 0.96, *P* < 0.05 from pre-Tx to intra-Tx weeks 1-3).

**Table 2.** Summary of neck nodal metastases tumor volume obtained with SMIT model and manual contouring on T_2_ weighted MR images for OPSCC patients.

Method	SMIT		Manual	
Statistics	Median [min, max] (cm^3^)	Mean ± SD (cm^3^)	Median [min, max] (cm^3^)	Mean ± SD (cm^3^)
Pre-Tx (week 0)	7.08	8.38 ± 7.01	7.65	8.68 ± 7.15
	[1.99, 33.21]		[1.70,33.97]	
Intra-Tx week 1	4.56	6.67 ± 5.44	5.95	6.87 ± 5.38
	[2.67, 24.68]		[1.88,23.37]	
Intra-Tx week 2	4.55	5.14 ± 3.32	4.66	5.68 ± 4.04
	[0.91,11.86]		[0.61,13.39]	
Intra-Tx week 3	4.35	5.24 ± 5.39	4.27	6.14 ± 5.82
	[0.28,18.71]		[0.36,17.67]	

SMIT and manual delineated longitudinal tumor volumes were not significantly different (*P* > 0.05).

**Figure 5.**
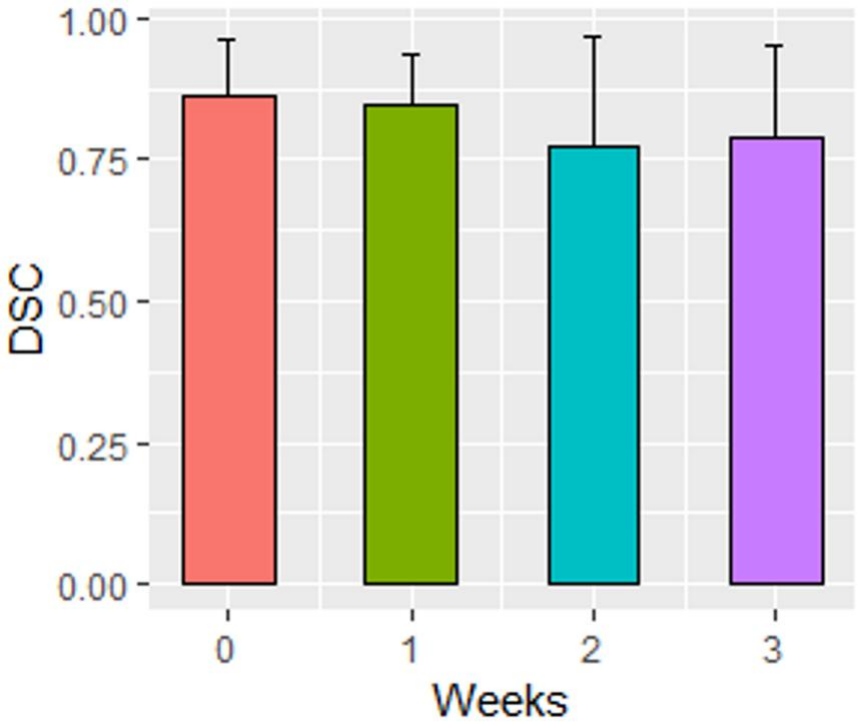
Bar plot comparing dice similarity coefficient (DSC) metric value for self-distilled masked image transformer (SMIT) auto-segmentation with reference to manual contouring for neck nodal metastases from oropharyngeal squamous cell carcinoma patients. The DSC values between treatment weeks were not significantly different (*P* > 0.05). Error bars are the standard deviation of the mean.

Mean longitudinal DSC values ranged from 0.86 to 0.79 (Table 3, Figure 5). DSC metric value was strongly positively correlated with volumes from the SMIT (*ρ* = 0.68, *P* = 0.003) and manual segmentation (*ρ* = 0.56, *P* = 0.02) at the intra-Tx week 1. Meanwhile, the DSC value showed a trend toward a positive correlation with the SMIT method and manually delineated tumor volumes at pre-Tx and intra-Tx weeks 2 and 3 (*P* > 0.05). The segmented Tx tumor volumes by both approaches were not statistically significant, as exhibited by DSC (*P* > 0.05).

**Table 3.** Dice similarity coefficient for performance of SMIT-based auto-segmentation compared with ground truth delineation.

Treatment week	Median	Mean ± SD
	[min, max]	
Pre-Tx (week 0)	0.89	0.86 ± 0.10
	[0.51, 0.95]	
Intra-Tx week 1	0.89	0.85 ± 0.09
	[0.59,0.94]	
Intra-Tx week 2	0.82	0.77 ± 0.19
	[0.22,0.95]	
Intra-Tx week 3	0.81	0.79 ± 0.16
	[0.33, 0.92]	

**Figure 6.**
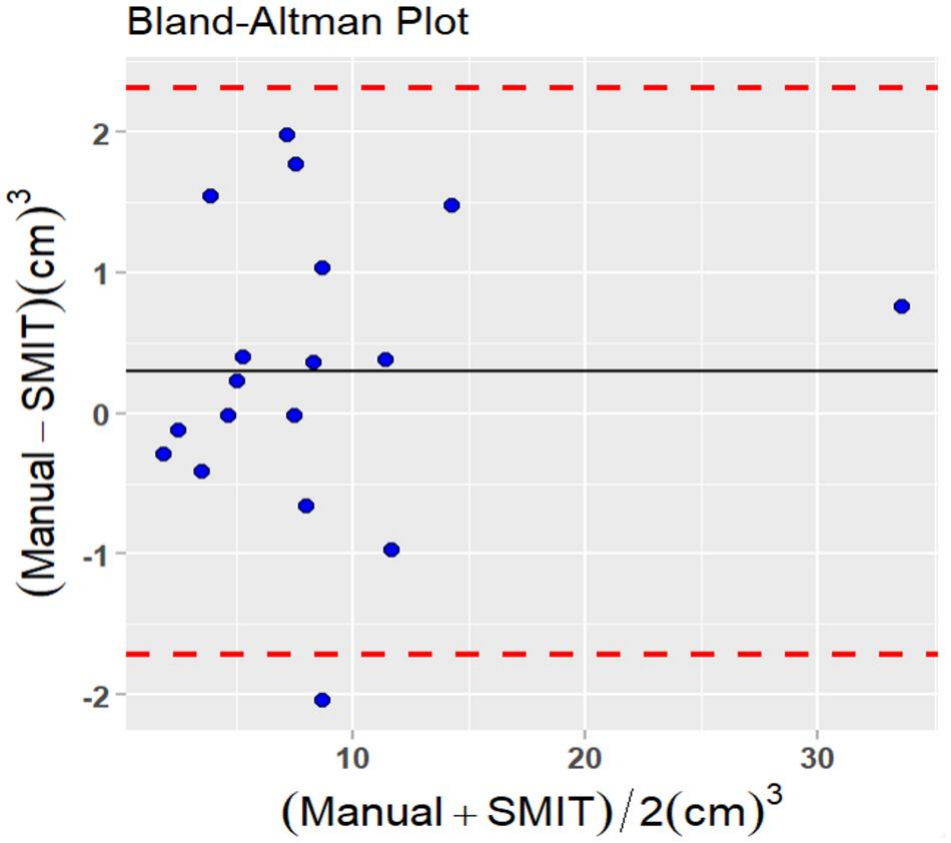
Bland-Altmann plot exhibiting for agreement between the measurements of nodal metastases mean total tumor with SMIT auto-segmentation model and manual contouring. Solid black lines are the mean difference (Δ) between the 2 measurements, and dash lines (red) are the 95% limits of agreement (mean Δ ± 1.96 SD).

The limits of the agreement (95%) were –1.71 to 2.13 cm^3^, with one outlier at pre-Tx shown in the Bland Altman plot (Figure 6). The mean difference in tumor volume (ΔV_0_) at pre-Tx between the SMIT method and manually delineated measurements was 0.3 cm^3^, and the mean differences in intra-Tx volumes (ΔV_1,2,3_) were 0.2,1.13, and 0.89, respectively.

## Discussion

We implemented and evaluated the segmentation accuracy of the SMIT method for neck nodal metastases on the longitudinal T_2_w MR images in 123 HPV+ OPSCC patients enrolled in the 30 Gy dose escalation clinical trial. This study compared the performance of the SMIT method to that of human experts. An advantage of using pretrained SMIT on longitudinal T_2_w MRI was that it could be fine-tuned with relatively few labeled image sets,^36^ enabling its application to modest HPV+ OPSCC patient datasets. There were no significant differences in volumetric measurements between contours generated by auto-segmentation or by a human expert, and the total tumor volume indicated by each was strongly correlated. The accuracy of the contours yielded by the SMIT algorithm was promising, and this level of performance indicates its potential for use in clinical radiation oncology HNC practice.

Time-consuming manual delineation of tumors on longitudinal MR images can hamper the development of adaptive radiotherapy treatment planning in patients with HNC.^39^ Previously, Walker et al^15^ published the largest prospective randomized controlled study evaluating the ROI accuracy and time-efficiency using atlas-based auto-segmentation software for OAR in the HNC RT planning. Atlas-based auto-segmentation saves delineation time in designing personalized strategies, but physicians’ approval remains vital for all OAR. Thus, a reliable and time-efficient method with less inter-reader variability is needed for tumor delineation in HNC RT clinics.

Several CNN-based algorithms have provided OAR and tumor volume segmentation and may need validation before use in RT clinics.^19^^,^^20^^,^^40^^,^^41^ 3D CNNs improved performance over 2D CNNs for MRI image segmentation tasks but place high demands on computational memory.^42^ Results demonstrated that cascaded 3D U-net CNNs could generate high-resolution segmentation with improved geometric accuracy. Ye et al^43^ applied dense connectivity embedding U-net CNN on multi-contrast MRI images (T_1_w and T_2_w) in 44 nasopharyngeal carcinoma patients. Results demonstrated that T_1_w and T_2_w MR combined images performed better (DSC = 0.72) than the single contrast (DSC = 0.64 for T_2_w and 0.62 for T_1_w). Lin et al^22^ evaluated the performance of the 3D CNN architecture of the VoxResNet model using multicontrast MRI images (T_1_w, T_2_w, postcontrast T_1_w, and fat-suppressed T_1_w) from 1021 nasopharyngeal cancer patients. The 3D CNN contours demonstrated a high level of accuracy when compared with ground truth contours testing in an independent dataset of 203 patients (DSC = 0.79). Wei et al^28^ used a slice-based interactive deep-learning (iDL) segmentation tool to evaluate the improvement of auto-segmentation accuracy with limited input from observers in 204 HNC patients, although their iDL approach was limited only to a few slices. Median segmentation accuracy at baseline was DSC = 0.65. Schouten et al^23^ reported disagreement in MV-CNN auto-segmentation and manual contouring mean primary tumor volumes in HNC (11.8 ± 6.70 cm^6^ 22.8 ± 21.1 cm^3^). In the present study, the SMIT auto-segmentation achieved lower DSCs on 2 patients who experienced a sharp reduction in node volumes during the treatments: one patient during intra-treatment week 2 (DSC = 0.22) and a different patient in week 3 (DSC = 0.33) (Table 3). For example, in the patient with week 2 DSC of 0.22, metastatic lymph node volume measured with SMIT and manual were 0.91 and 2.05 cm^3^, while the pre-treatment SMIT and manual volume were = 33.21 cm^3^ vs manual = 33.97 cm^3^, and DSC of 0.87. Notably, the DSC metric tends to exhibit higher DSC values for larger volumes and lower DSC values for smaller volumes. This principle was also demonstrated by Schouten et al.^23^ Initial CNN-based auto-segmentations, including 3D Unet, MV-CNN, interactive deep-learning (iDL), and 3D Residual U-net, showed promise for HNC^23^^,^^24^ but their performance, time efficiency, and ease of use need to be validated prior to their application for RT planning.

Emerging SSL vision transformers have shown promising results in specific tasks, but they may not necessarily be superior to CNNs in all scenarios.^36^^,^^44^ The choice between SSL vision transformers and CNNs depends on several factors, including the specific task, the amount of available training data, and computational resources.^44^ Evaluating the strengths and weaknesses of each approach is an active research topic. To our knowledge, only one prior study has used vision transformer-based models for segmenting primary HN tumors from CT and fluorodeoxyglucose (FDG)-positron emission tomography (PET) images. Sobirov et al^45^ used vision transformer-based models for segmenting primary HN tumors from CT and FDG-PET images and achieved a mean DSC of 0.736(±0.043). Ours is the first study using the SMIT method for auto-segmentation of neck nodal metastases with longitudinal T_2_w MR images obtained from HPV+ OPSCC patients and exhibiting concordance between this method and ground truth.

Our study has a few limitations. All scans were acquired under the same prospective IRB protocol using a single MRI scanner at a single institution. The next step to address this limitation is external validation on an independent test set of HNC patients. Demonstrating the robustness of our algorithm to common sources of variation in images acquired at different clinical sites will be an important step toward its translation to help clinicians with segmentation tasks. For wider imaging community use, we are making our method freely available to the research community through GitHub to support open-source research.

## Conclusion

In conclusion, the SMIT method demonstrated promising performance for auto-segmenting tumor volume compared to ground truth delineations before and during intra-Tx in HPV+ OPSCC patients. Our auto-segmentation method could be valuable for improving the segmentation efficiency, reducing inter-operator variability, and facilitating the reliability of tumor delineations in RT clinics for HNC patients.
